# Quantitative magnetic resonance imaging assessment of muscle composition in myotonic dystrophy mice

**DOI:** 10.1038/s41598-023-27661-w

**Published:** 2023-01-10

**Authors:** Ariadna Bargiela, Amadeo Ten-Esteve, Luis Martí-Bonmatí, Teresa Sevilla, Manuel Perez Alonso, Ruben Artero

**Affiliations:** 1 Neurology Department, La Fe Health Research Institute (IISLAFE), Neuromuscular Research Unit, Valencia, Spain; 2 Biomedical Imaging Research Group (GIBI230) and “La Fe” Imaging Node of the Distributed Biomedical Imaging Network (ReDIB), Singular Scientific and Technical Infrastructures (ICTS), Valencia, Spain; 3 Medical Imaging Department, La Fe University and Polytechnic Hospital, Valencia, Spain; 4 Centro de Investigación Biomédica en Red de Enfermedades Raras (CIBERER), Valencia, Spain; 5 Department of Medicine, Universitat de València, Valencia, Spain; 6 University Research Institute for Biotechnology and Biomedicine (BIOTECMED), University of Valencia, Valencia, Spain; 7 Translational Genomics Group, INCLIVA Biomedical Research Institute, Valencia, Spain

**Keywords:** Mutation, RNA splicing, Experimental organisms, Imaging

## Abstract

Myotonic dystrophy type 1 (DM1) is a severe autosomal dominant neuromuscular disease in which the musculoskeletal system contributes substantially to overall mortality and morbidity. DM1 stems from a noncoding CTG trinucleotide repeat expansion in the *DMPK* gene. The human skeletal actin long repeat (HSA^LR^) mouse model reproduces several aspects of the disease, but the muscle-wasting phenotype of this model has never been characterized in vivo. Herein, we used quantitative MRI to measure the fat and muscle volumes in the leg compartment (LC) of mice. These acquired data were processed to extract relevant parameters such as fat fraction and fat infiltration (fat LC/LC) in HSA^LR^ and control (FBV) muscles. These results showed increased fat volume (fat LC) and fat infiltration within the muscle tissue of the leg compartment (muscle LC), in agreement with necropsies, in which fatty clumps were observed, and consistent with previous findings in DM1 patients. Model mice did not reproduce the characteristic impaired fat fraction, widespread fat replacement through the muscles, or reduced muscle volume reported in patients. Taken together, the observed abnormal replacement of skeletal muscle by fat in the HSA^LR^ mice indicates that these mice partially reproduced the muscle phenotype observed in humans.

## Introduction

Myotonic dystrophy type 1 (DM1) is a debilitating chronic disease caused by an expansion of the unstable CTG triplet repeat in the 3' untranslated region of the *DM1 protein kinase (DMPK)* gene. DM1 is autosomal dominant and can affect all ages, from newborns to elderly adults. Although DM1 is the most common form of muscular dystrophy in adults and the prevalence of the mutation is 1/2100 births^1^, there are currently no treatment options for this disease. Adult-onset patients face risks of physical disability and a shortened lifespan. Characteristic neuromuscular symptoms include muscle stiffness and weakness, impaired muscle relaxation capacity (myotonia), and progressive loss of muscle mass (atrophy). However, DM1 is not merely a skeletal muscle disease but a multisystemic disease, as other tissues and organs, such as the smooth muscle, brain, and heart, are also affected^2^. Premature deaths among DM1 patients are caused by respiratory failure due to muscle weakness and myotonia that affect the peripheral respiratory system. Additionally, failure of the central respiratory drive and the upper airway muscles contributes to an increased risk of pulmonary infections in patients^3,4^.

Muscle atrophy and wasting are critical signs of DM1, and various factors have been hypothesized to lead to these phenotypes^5^. The principal factor consists of *DMPK* transcripts carrying the DM1 mutation. Mutant RNA is retained in the cell nucleus in several tissues, including the heart, brain, and skeletal muscle, where it aggregates into insoluble, stable, membraneless structures known as foci. Ribonuclear foci of expanded CUG repeat hairpins can bind and sequester RNA-binding proteins, thus preventing them from performing their normal functions^6^.

Magnetic resonance imaging (MRI) is the gold-standard technique in muscle pathology. The benefits include its capacity to discriminate muscular from adipose tissue accurately and to identify fat infiltration into skeletal muscle^7,8^. Consequently, this technique helps diagnose neuromuscular pathologies, as it can reveal disease-specific patterns of muscle involvement^9^. Furthermore, as MRI is a noninvasive technique that does not expose patients to ionizing radiation, it can be used to track the evolution of muscular diseases. In musculoskeletal disorders, MRI is being used increasingly often for longitudinal evaluation of candidate drugs^10^ and validation of clinical trial outcomes^11^.

Clinical research on DM1 has categorically demonstrated that patients display muscle defects that can be quantitatively analyzed using MRI. Specifically, DM1 patients have almost 4 times more fatty infiltration in the skeletal musculature than unaffected controls. Additionally, fat infiltration correlates with scores on the 6-min walk test and the Muscular Impairment Rating Scale^12^. Patients also show significantly reduced skeletal muscle volume, especially in the gastrocnemius medialis and soleus, accompanied by increased T2 water relaxation time, indicating muscle atrophy and edema, respectively^12,13^. Other MRI studies have shown that over 70% of DM1 patients display fatty infiltration in the lower extremities, with especially severe defects in the gastrocnemius and tibialis anterior during the initial stages of the disease^14^. Interestingly, the authors reported the possibility of detecting affected muscles even before weakness was clinically noted. Recent studies have shown that an increased fat fraction in the muscles of DM1 patients is correlated with muscle weakness, reduced specific strength, and high disease severity^15,16^. Taken together, these data support the idea that muscle impairment as determined by quantitative MRI is strongly correlated with strength, supporting its feasibility as an approach to predict muscle dysfunction in DM1.

Nearly two dozen experimental treatments against DM1 are in development^17^, but there are insufficient noninvasive methods for preclinical evaluation of these candidate drugs in DM1 model mice; such methods would allow dynamic evaluation of phenotype progression. Therefore, we decided to optimize quantitative MRI to these circumstances and present an in vivo method to quantitatively measure muscle defects in a murine model expressing 250 CUG repeats in the skeletal muscles, driven by a human skeletal actin transgene^18^ (HSA^LR^). The HSA^LR^ murine model accurately reproduces key DM1 phenotypes such as aberrant alternative splicing, centrally located nuclei in muscular fibers, variable muscle fiber size, and myotonia^18–20^. Some further studies performed in young HSA^LR^ mice have reported that chemical inhibition of glycogen synthase kinase 3 beta (GSK3β) corrects impaired Celf1 activity, thus preventing the development of DM1 muscle pathology, including muscle atrophy^21^. However, independent studies have reported a lack of clear and consistent dystrophic and atrophic features in this model^18,19^. Conversely, researchers have discovered genetic and drug manipulations that can enhance muscle atrophy phenotypes in HSA^LR^ mice^22,23^. Selecting the disease-related phenotypes already described in DM1 patients, our study aimed to achieve MRI quantitation of muscle and fat volume, fat infiltration, and fat fraction (FF) in HSA^LR^ model mice.

## Results

### MRI acquisition in DM1 model mice

**Figure 1:**
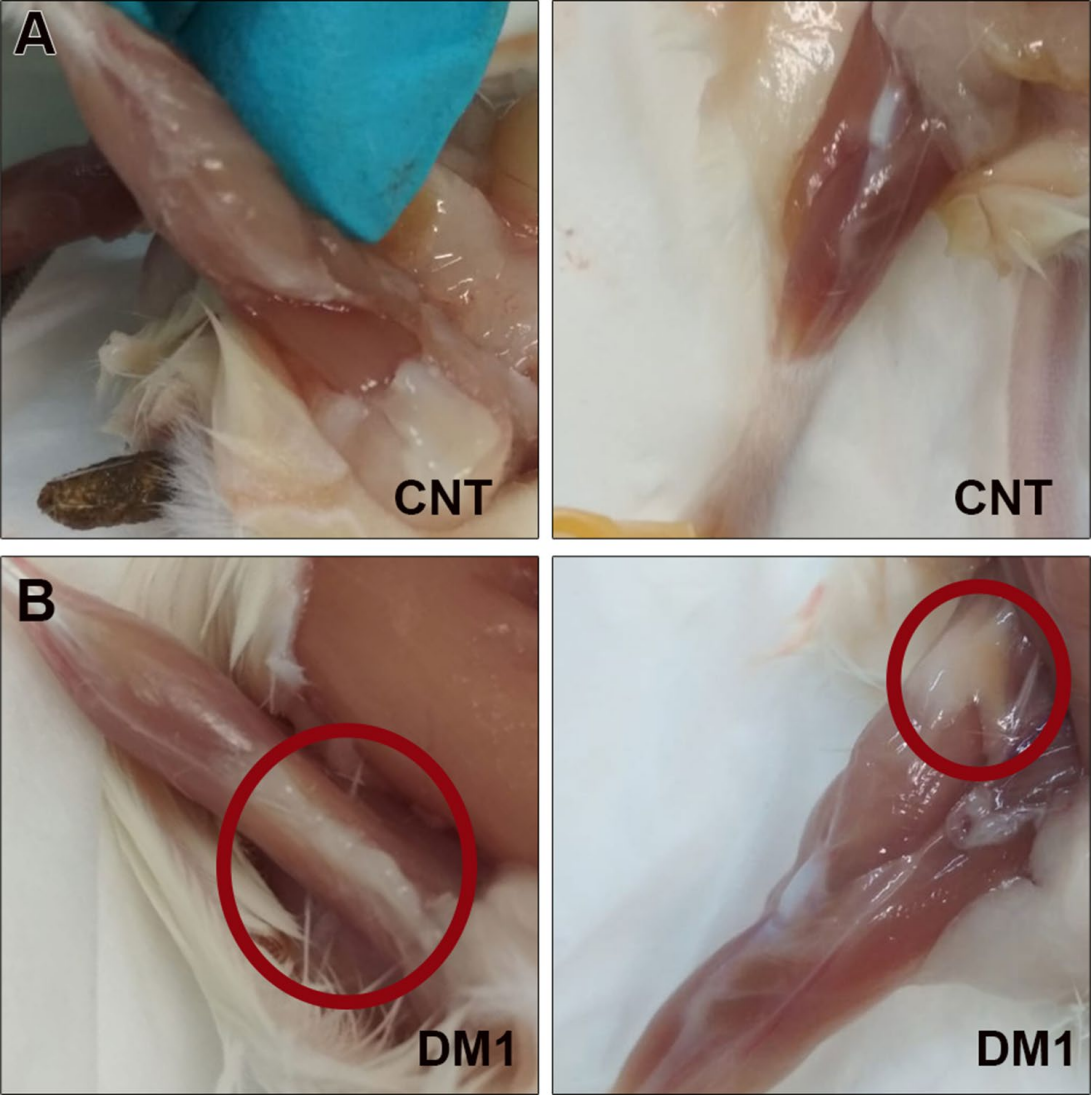
Skeletal muscle is replaced by fatty tissue in HSA^LR^ mice. Representative images of dissected hind legs from control (**A**) and DM1 model mice (**B**). Red circles denote visible infiltrated fat in the gastrocnemius (left) and quadriceps (right) muscles of model mice.

**Figure 2:**
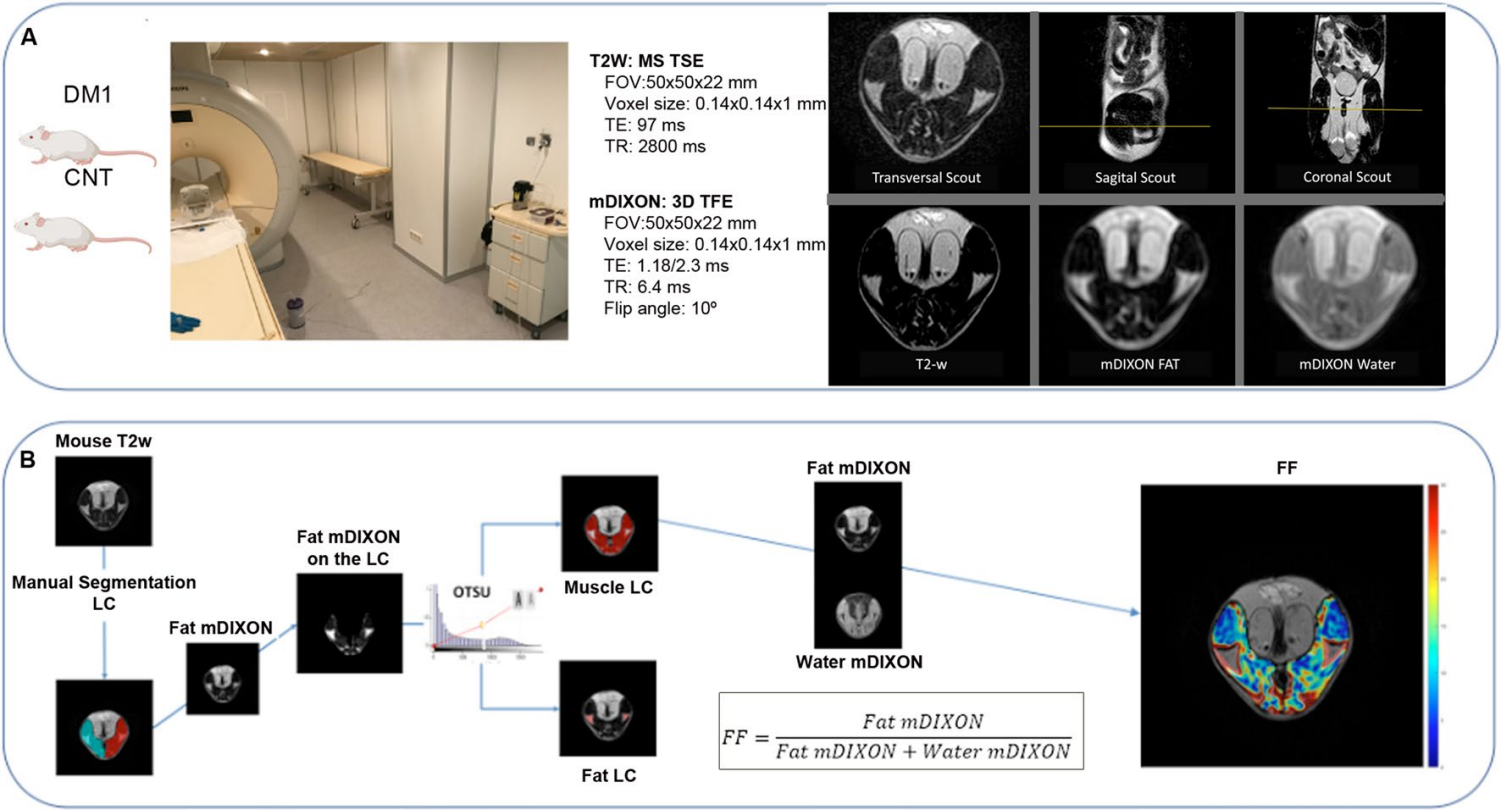
Summary of image acquisition and analysis workflow. The image depicts the infrastructure used for mouse sedation and MRI acquisition, showing both the angulation and the main parameters of the sequences used to obtain the cross-sectional images of a DM1 model mouse thigh (**A**), along with the workflow of muscle compartment segmentation, the automatic segmentation of fat and muscle, and the postprocessing used to obtain the muscle/fat fraction mapping (**B**). *LC* leg compartment, *FF* fat fraction. Figure partially created with BioRender.com.

Necropsies of 4.5-month-old HSA^LR^ mice showed clear signs of fatty infiltration in the quadriceps and gastrocnemius muscles, as fatty clumps could easily be observed (Fig. 1). To quantitatively analyze these phenotypes in vivo, images from eleven male HSA^LR^ mice and eleven age-matched counterpart control (FVB) mice were acquired under anesthesia in an MR scanner. Two individuals from each group had to be removed from the study because their breathing movement impeded the acquisition of images of sufficient quality for further quantitative analyses. Images of both hind limbs from each animal were acquired, and the leg compartment (LC) was segmented manually. The LC consists of all muscles, fat, and bones forming the leg, but the small size of the mice compared to the scanners used in clinical practice precluded us from differentiating small bones from muscle; therefore, the LC was stratified into macroscopic fat (fat LC) and muscle plus bone, defined as muscle LC. Note that, as the animals used in the experiment were of the same sex and age and did not express the transgenic construct in bone or bone precursors, bone structure size and density were assumed to be constant among the different individuals. A schematic workflow of the process is shown in Fig. 2.

### Different MRI outcome measures between DM1 model mice and controls

**Figure 3:**
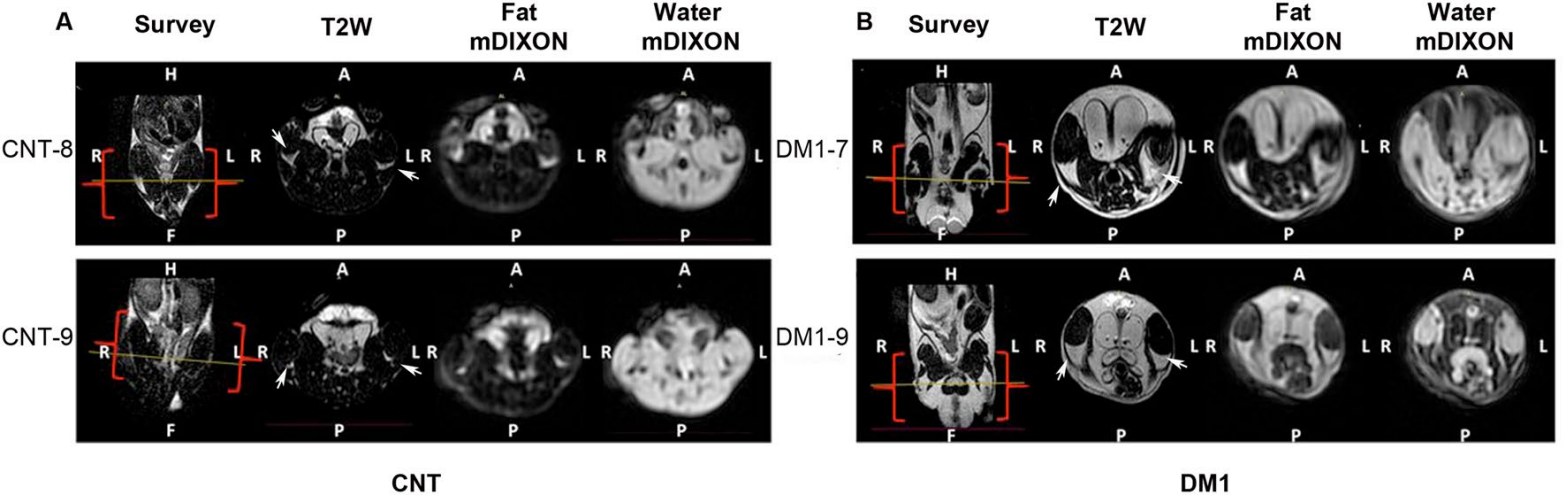
DM1 model mice show partial pathological phenotypes. Representative axial images from T2W, fat and water mDIXON of two control (**A**) and two DM1 model mice (**B**). The position is indicated on the coronal survey, and the red square brackets delimit the coverage of the acquired volume. *H* head, *F* foot, *A* anterior, *P* posterior, *R* right, *L* left. Arrows point to fat in the LC.

**Figure 4:**
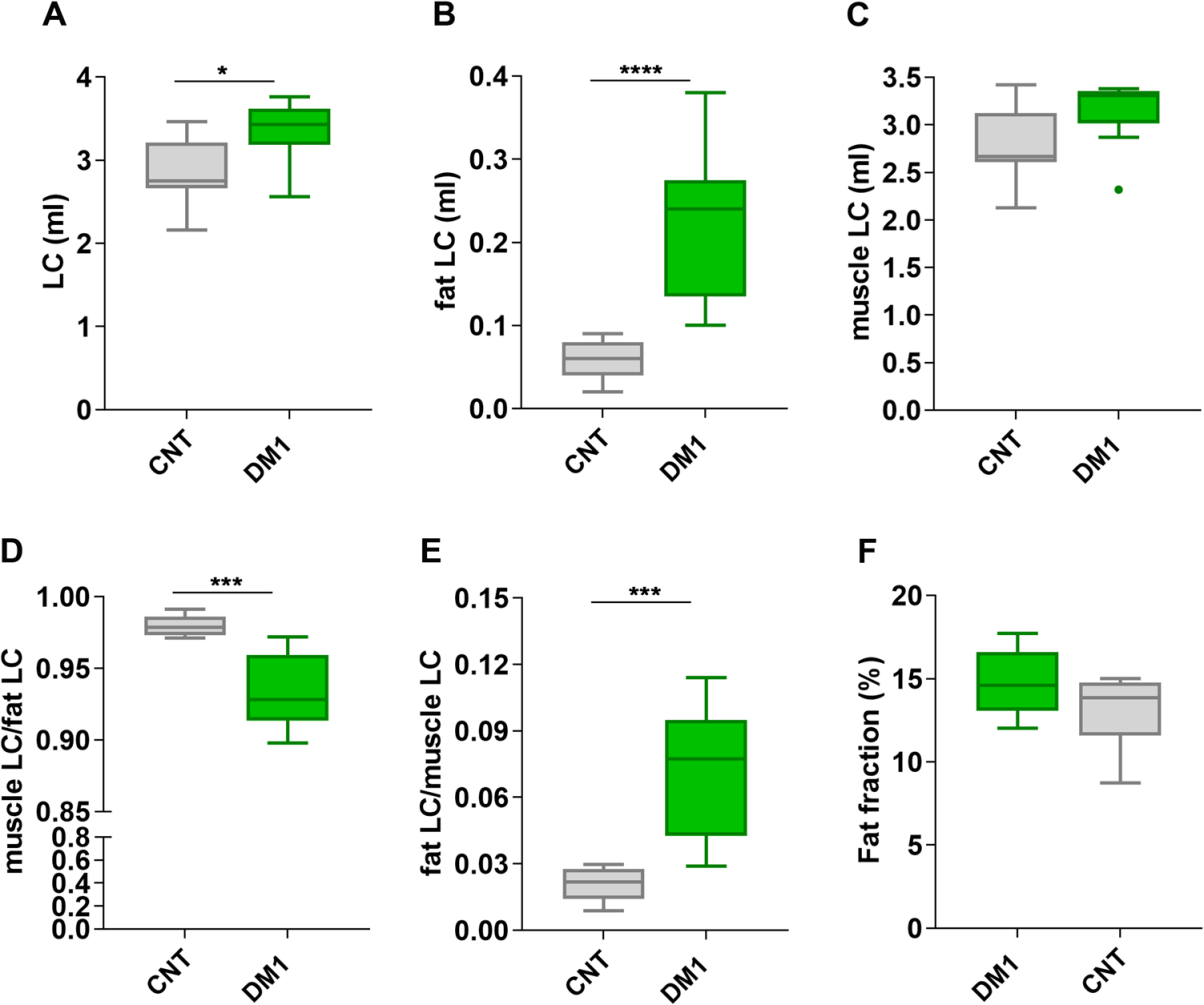
HSA^LR^ mice display fat infiltration in skeletal muscle. Plots represent the total volume (**A**), the volume of fat (**B**), and the volume of muscle (**C**) in the leg compartments of control (CNT) and DM1 model mice. Values were obtained from quantitative analyses of MRI data (n = 9 mice per group). Muscle-to-fat ratios (**D**) were also calculated. Disease model mice showed increased fat infiltration levels in skeletal muscle (**E**). No differences in the fat fraction were detected between control and model mice (**F**). The boxes extend from the 25th to the 75th percentile, the line in the middle of the box is plotted at the median, and the whiskers reach down to the smallest value and up to the largest. *p < 0.05, ***p < 0.001, ****p < 0.001 according to a 2-tailed Mann‒Whitney *U* test.

Image analysis from manual segmentation provided the whole LC volume of both hind legs, corresponding to the combined muscle LC and fat LC (Table 1, Fig. 3A,B). DM1 model mice had significantly higher LC volume (18%) than the control group (Fig. 4A), consistent with the observed greater weight of DM1 mice. Specifically, the mean weight of control FVB mice (CNT) (n = 14) was 27.9 g vs. 30.71 g for DM1 (n = 20) (p = 0.0423; Mann‒Whitney *U* test). Individual analysis of both LC components showed that the difference was mainly due to a robust, almost fourfold increase in fat LC volume in the DM1 group relative to controls (Fig. 4B). In contrast, no statistically significant difference in muscle LC volume was detected between the two experimental groups (Fig. 4C).

**Table 1:** Quantitative values from MRI measures in control (CNT) and HSA^LR^ (DM1) mice.

	Muscle compartment (ml)	Fat (ml)	Fat fraction (%)	Fatty infiltration
CNT 1	3.14	0.09	14.99	0.029
CNT 2	2.75	0.08	13.86	0.029
CNT 3	3.28	0.08	14.72	0.025
CNT 4	2.66	0.05	14.68	0.019
CNT 5	2.80	0.06	14.82	0.021
CTN 6	2.66	0.06	13.22	0.023
CNT 7	3.46	0.05	8.75	0.013
CNT 8	2.16	0.03	11.34	0.015
CNT 9	2.68	0.02	11.83	0.009
DM1 1	3.28	0.11	14.59	0.035
DM1 2	3.62	0.24	17.40	0.066
DM1 3	3.09	0.22	17.73	0.072
DM1 4	2.56	0.24	13.61	0.095
DM1 5	3.41	0.10	12.03	0.028
DM1 6	3.43	0.27	12.53	0.078
DM1 7	3.61	0.28	15.77	0.078
DM1 8	3.49	0.16	15.12	0.047
DM1 9	3.76	0.38	14.08	0.102

Several fat LC and muscle LC ratios in the LC were generated to determine the relative contributions of each tissue fraction to the disease phenotype. Specifically, the muscle LC/fat LC ratio revealed that although the muscle LC fraction did not differ significantly from controls in volume, its ratio to the amount of fat was significantly reduced in DM1 mice (Fig. 4D). Similarly, fatty infiltration in the LC was calculated as the ratio of fat LC to total LC, and the results confirmed that HSA^LR^ mice had more than threefold higher fatty infiltration levels than FVB control mice (Fig. 4E). For quantitative determination of the muscle fat fraction, we employed the fat and water images provided by the mDIXON sequence over the voxels corresponding to the muscle LC. These analyses revealed that the model mice did not reproduce the increased fat fractions observed in humans^12,13,15^ (Fig. 4F), despite a trend-level increase.

## Discussion

Muscle MRI has recently become a prominent diagnostic procedure in neuromuscular disorders^24^. Recent studies have shown that DM1 patients have several affected muscle parameters that can be quantitatively analyzed by this technique^13–15,25^. These alterations include fat infiltration, fat fraction, reduced muscle mass, muscle atrophy, edema, impaired contractile muscle volume, and altered muscle size. In this work, we sought a more in-depth characterization of a murine model widely used by the scientific community by using MRI to investigate whether it reproduces the muscle defects detected in patients. Some studies have already suggested that HSA^LR^ mice are somewhat limited in robustly reproducing muscle atrophy phenotypes^18,23^. Quantitative analyses of MRI data have confirmed that these mice do not reproduce all the muscle-wasting-related alterations described in recent years in DM1 patients. Specifically, while an increased fat and leg volume ratio were found, analogous to findings in patients, a normal fat fraction was detected. Interestingly, model mice have large lumps of adipose tissue within limb skeletal muscles, whereas in patients, fatty infiltration is more dispersed and heterogeneous^12,13,16^. In other words, the human phenotype is quantitatively reproduced in mice, but significant qualitative differences exist. However, parameters related to muscle atrophy remain unaltered in model mice, as no difference in muscle LC volume or fat fraction was observed between HSA^LR^ mice and the control group. Fat fraction is a key parameter in the context of the disease, given that, in addition to being increased in patients, it strongly correlates with disease severity and progression^12,16^, but we detected no significant differences in this measure. Interestingly, HSA^LR^ mice showed higher body weight and fat volume than controls, in agreement with what is observed in DM1 patients^26^.

Increased activity and stability of GSK3β have been proposed to explain the atrophic phenotype in DM1^21,27^. In recent years, however, additional molecular alterations have been found to contribute to the phenotype^5^. Pathogenic upregulation of Musashi 2 (MSI2) in DM1 cells, which inhibits miR-7 biogenesis, leads to increases in autophagy and the activity of the ubiquitin‒proteasome system, two pathways with a strong contribution to the muscle impairment characteristic of DM1^28–32^. Nonetheless, miR-7 levels in the diaphragm and gastrocnemius of HSA^LR^ mice were similar to those of control mice^29^. Therefore, the failure to reduce miR-7 in model mice may explain why no overt muscle atrophy was detected.

Preclinical testing of therapeutic effects in a mammalian animal model is a requirement of any drug development procedure. No studies have yet been published in which MRI determinations have been performed in the widely used HSA^LR^ DM1 model mice^33–35^. Therefore, our finding that they reproduce some of the muscle-pathology-related phenotypes observed in patients, such as fat replacement, makes this model a valuable tool during preclinical evaluation. A particularly useful aspect of MRI in this context is that it is a noninvasive technique performed in vivo, enabling scientists to evaluate the effect of a candidate treatment on the same individual at different time points during the experiment. This brings two advantages: first, it allows researchers to study the phenotype's evolution over time; second, it allows them to identify the optimal treatment duration or point of maximum effect. Moreover, it allows the study of all muscles, rather than only a few, as with biopsy or electromyography.

In conclusion, we observed evidence of fatty infiltration in the leg muscles of DM1 mice in necropsies, and quantitative MRI confirmed this phenotype, which has been reported to affect more than 70% of DM1 patients^14,36^. Our results are valuable for therapy research due to the vast difference between control and DM1 mice, allowing researchers to identify molecules with strong therapeutic potential.

## Methods

### Animal experimentation

Mouse handling and experimental procedures were performed according to the European law regarding laboratory animal care and experimentation (2003/65/CE) and the Animal Research: Reporting of in vivo Experiments (ARRIVE) guidelines. All procedures were approved by the Consejeria de Agricultura, Generalidad Valenciana (Reference Number 2019/VSC/PEA/0198).

Eleven male homozygous transgenic HSA^LR^ mice (line 20b) were used as DM1 disease models; they were compared to 11 controls, which were mice with the same genetic background (FVB). Mice were provided by C. Thornton (University of Rochester Medical Center, Rochester, NY)^18^. All animals were 4.5 months old at the time of experimentation and were randomly selected for the experiment. The animals were bred in the animal facilities of the University of Valencia and then transferred to the facilities of the La Fe University Hospital. Once the acclimatization period had elapsed, images were acquired by MR while the animals were anesthetized with isoflurane and immobilized in the supine position; after acquisition was completed, the animals were sacrificed by cervical dislocation.

For the necropsy study, six HSA^LR^ and six FVB mice were euthanized by cervical dislocation. All animals were 4.5-month-old males. The procedure was approved by the Consejería de Agricultura, Generalidad Valenciana (Reference Number 2018/VSC/PEA/0182).

### MR acquisition

MR images were obtained using a Philips Achieva 3.0 TX (Amsterdam, The Netherlands) using an 8-Channel Sense Wrist Coil. Animals were placed in a prone position for transverse acquisition; the protocol was created with 22 slices to cover the full thigh. This plan was made on the basis of sagittal and coronal scout images. The acquisition protocol consisted of a T2-weighted multi-slice turbo spin‒echo (T2W MS TSE) sequence with a field of view (FOV) of 50 × 50 × 22 mm in the transverse plane (22 slices), voxel size = 0.14 × 0.14 × 1 mm; TE = 97 ms and TR = 2800 ms and an mDIXON sequence with the same FOV and voxel size; TE = 1.18/2.3 ms, TR = 6.4 ms and a flip angle of 10°. The mDIXON (modified Dixon) sequence employed in this study was developed by Philips researchers; this sequence employs two echoes with unrestricted TE values, a 7-peak fat model, and a B0 correction algorithm to improve fat-free imaging.

### Image processing

The purpose of the image processing was to identify fat LC and muscle LC, from which fat infiltration, defined by the ratio between fat LC to the total LC volume and by the fat fraction over muscle LC, was determined.

For this purpose, acquired images were converted from DICOM to NIFTI, and the LC was manually segmented in both muscles using open-access ITK-SNAP software^37^; two individuals from each group were excluded during this process due to excessive movement.

The Otsu method^38^ (an image processing method to separate voxels into two classes) was implemented in MATLAB (R2016b, MathWorks, Natick, MA) and applied to the fat image provided by the mDIXON sequence to separate muscle LC from fat LC in the regions delineated as LC. The fat fraction mapping was obtained employing the water and fat images provided by mDIXON over muscle LC voxels according to the following formula: $FF=\frac{fat\, mDIXON}{fat\, mDIXON+water\, mDIXON}$. This procedure is schematically represented in Fig. 2. LC, muscle LC, and fat LC volumes were obtained from the sum of voxels belonging to each region and voxel size, and these and other ratios between these regions are presented in the results section (Table 1 and Fig. 4); among these ratios, fat infiltration is highlighted as the ratio of fat LC to LC.

### Statistical analyses

The Pingouin 0.5.1 library developed in Python for image processing results was used for statistical analysis. First, the Shapiro–Wilk test and Levene’s test, respectively, were applied to determine the normality and homoscedasticity of the distributions. ANOVA tests were performed using ANOVA for comparisons if both groups showed homoscedasticity and Welch’s ANOVA otherwise. In cases where the samples of either group did not follow a normal distribution, differences between control and DM1 model mice were assessed with a 2-tailed Mann‒Whitney *U* test.

## Data Availability

The datasets used and analyzed during the current study are available from the corresponding author upon reasonable request.
